# Fine-tuning Metabolic Switches

**DOI:** 10.1371/journal.pbio.1001664

**Published:** 2013-09-24

**Authors:** Caitlin Sedwick

**Affiliations:** Freelance Science Writer, San Diego, California, United States of America

Cells get the energy for most of their activities in the form of adenosine triphosphate (ATP), which is generated through the breakdown of glucose and lipid molecules. Partial breakdown of a single glucose molecule through glycolysis yields 2 ATP molecules; and when glycolysis is followed by pyruvate decarboxylation and the tricarboxcylic acid (TCA) cycle, a cell can eke out between 30 and 38 ATP molecules per molecule of glucose. Lipids are another potent energy source that, after some preliminary processing, can also be metabolized through the TCA cycle.

Disruptions to metabolic conditions (such as changes in environmental oxygen availability) are dangerous to many organisms, because while glycolysis can occur in the absence of oxygen the TCA cycle cannot. Glycolysis isn't sufficient to meet the long-term energy needs of, for example, the adult human heart, which consumes large amounts of ATP for each of the more than 6 billion beats it undergoes in the average lifetime. But fine-tuning of the glycolytic pathway can be critical to meet cells' energy needs in certain scenarios, as a pair of papers published in this month's *PLOS Biology* demonstrate.

A paper by a multinational team of researchers headed by Ross Breckenridge and Timothy Mohun at the MRC-London investigates a metabolic switch that occurs in the hearts of neonates. The second paper, by Tobias Eckle, Holger Eltzschig, and colleagues at the University of Colorado, Denver, looks at the metabolic changes that accompany acute lung injury. Although the research teams had different aims and were studying different tissues and biological problems, the efforts of both groups have highlighted how the regulation of metabolic responses can affect clinical outcomes.

In point of fact, glycolysis can suffice for the energy needs of some tissues; it's been known for some time that the fetal heart relies exclusively on glycolysis in the low-oxygen environment of the womb. In the heart, therefore, oxidative pathways only come into play after birth. How do heart cells pull off this metabolic switch? We now have a much better grip on this process thanks to the paper by Breckenridge et al.

Breckenridge and colleagues were interested in a protein called Hand1, a transcription factor that is abundantly expressed in embryonic but not adult cardiomyocytes. If Hand1's expression changes around the time of birth, the group reasoned, then it's possible Hand1 might be involved in the observed metabolic switch. Indeed, they found that Hand1 levels drop dramatically soon after birth, because Hand1's expression is turned on by a protein called hypoxia-inducible factor-1α (HIF1α). And HIF1α, as its name implies, is expressed only under low oxygen conditions.

To see how Hand1 expression impacts heart metabolic pathways, the researchers used microarray analyses to compare gene expression patterns in normal hearts to the patterns in hearts of mice that overexpress Hand1. They showed that Hand1 specifically represses the expression of proteins involved in lipid metabolism and in the mitochondrial TCA cycle, often by directly binding to and repressing the genes' promoters.

**Figure 1:**
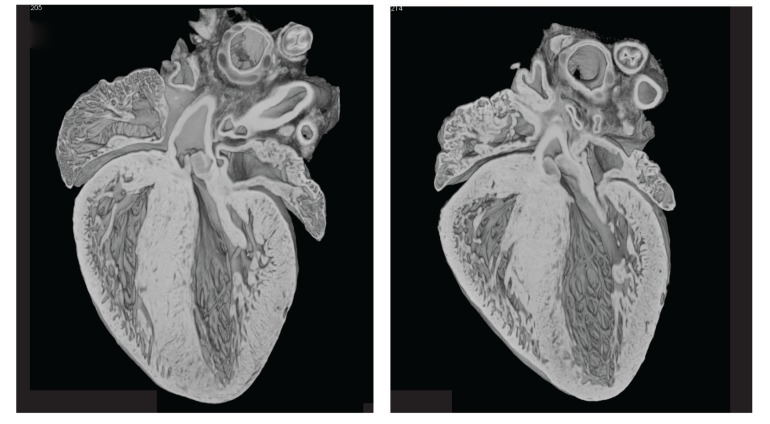
Sectioning and reconstruction of control heart (left) and Hand1-overexpressing heart (right) show no obvious structural defects, but Hand1-overexpressing mice die soon after birth.

After birth it's important for the neonatal heart to turn off Hand1 expression so that the tissue can start using the more efficient oxidative metabolic pathways (Figure 1). Consequently, mice overexpressing Hand1 exhibit respiratory distress and high death rates soon after birth; they are relying on glycolysis, which by itself can't generate enough energy for the adult organ's metabolic needs. But that's not to say that Hand1 finds no use in adult life; in fact, the authors note that Hand1—and therefore glycolytic metabolism—is re-expressed in the hearts of adults undergoing heart failure. The authors think this may represent cells' last-ditch efforts to adapt to the chronic ischemic conditions that assail the failing heart.

So glycolysis is important in chronic heart failure, but what about acute ischemic insults, such as occur during myocardial infarcts? Might the Hand1 pathway step in to divert metabolic pathways when oxygen suddenly isn't available? Curiously, even though HIF1α expression does increase under these conditions, Hand1 expression does not. Nonetheless, the authors found that mutant hearts overexpressing Hand1 showed enhanced tolerance to acute ischemic insults. Therefore, a metabolic switch to glycolysis can help support cell function under less-than-ideal conditions. It may be useful to investigate this in medical scenarios such as myocardial infarction.

Importantly, Eckle et al. also demonstrated the importance of tissues' ability to remodel their metabolic pathways, albeit in a different context: while looking at acute lung injuries (ALI) of the type that can occur during major surgery. ALI can result in inflammation, pulmonary edema, impaired lung-blood gas exchange, and in severe cases it can be lethal. If survived, severe ALI can have lasting negative impacts on patients' quality of life. But ALI also frequently resolves itself without any long-term effects, and Eckle et al. were interested in how this might happen.

ALI is usually caused by lung infections, but it can also result when patients are put on mechanical ventilators (which force air into the lungs of people who cannot breathe on their own). It's thought that the cyclic mechanical stretching caused by ventilator machines causes stretch-induced injuries to lung tissue. On the other hand, mechanical stretching may also induce protective pathways. So, to mimic the effects of ventilator-induced stretch, the authors grew lung epithelial cells on a repeatedly stretching, flexible surface. To find out what effects this treatment has on the cells, the authors used gene expression microarrays to investigate gene expression patterns.

**Figure 2:**
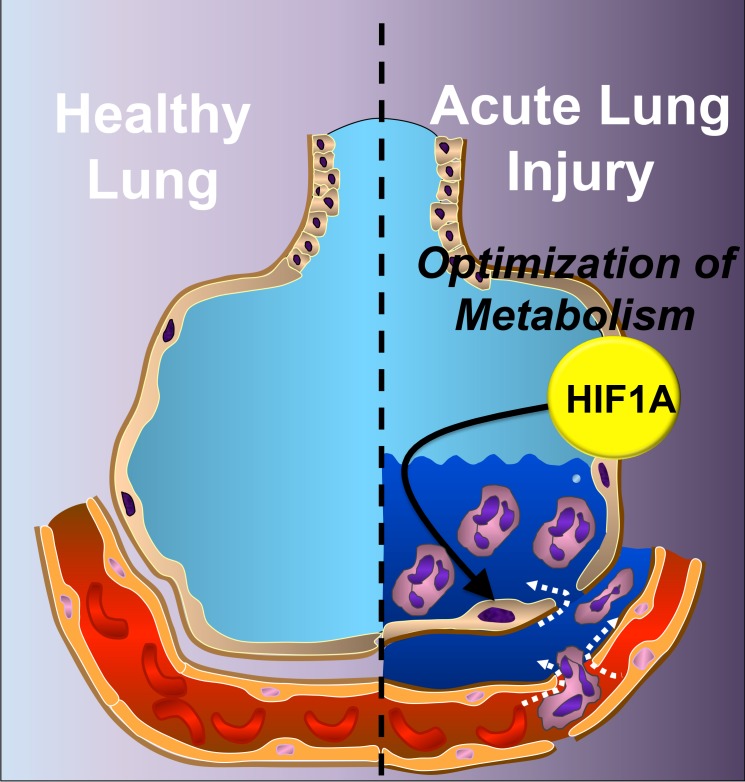
Acute lung injury (ALI)—one of deadliest diseases in critical care units—is associated with the stabilization of hypoxia-inducible factor-1α (HIF1α). HIF1α dampens lung inflammation and pulmonary edema and provides protection from injury by improving alveolar epithelial carbohydrate metabolism. *Image credit: Shelley A. Eltzschig*.

To their surprise, Eckle and colleagues observed that the gene expression pattern in mechanically stretched cells strongly resembled that observed in cells exposed to hypoxic conditions (Figure 2). Because HIF1α is well known to govern gene expression in response to hypoxia, the group looked at HIF1α levels in the stretched cells. They found that mechanical stretching somehow increases HIF1α expression even when cells aren't hypoxic.

Under normal oxygen levels (normoxic conditions), HIF1α expression levels are usually kept low via the rapid degradation of the protein. However, while it is unusual to observe HIF1α expression in normoxic cells, previous studies had shown that HIF1α degradation is prevented in cells lacking an enzyme called SDH. When Eckle and colleagues looked at SDH activity in their cells, they found that it is lower in mechanically stretched cells than in normal ones, explaining why HIF1α expression is stabilized in these cells.

The authors demonstrated that HIF1α stabilization causes strong upregulation of genes involved in glycolysis. Because this was taking place under normoxic conditions, the increased glycolytic activity was also accompanied by increased TCA cycle activity and therefore increased ATP production. Later experiments showed that these effects are not restricted to the cell culture model of ALI; similar effects are observed in the lungs of animals exposed to ventilator-induced ALI. Stretch-induced HIF1α stabilization is observed specifically in lung alveolar epithelial cells (but not in other cells, such as immune cells, that are also found in the lung).

What purpose does this metabolic shift serve? The researchers found that the normoxic stabilization of HIF1α and concomitant increases in glycolytic and TCA activity make lung tissue more resilient to the mechanical insults of ventilator-induced ALI. Lungs of mice lacking HIF1α in alveolar epithelial cells show increased edema and inflammation, while lungs of mice with additional, pharmacologically induced stabilization of HIF1α experience enhanced protective effects.

Taken together, the studies by Breckenridge et al. and Eckle et al. strongly demonstrate that fine-tuning of metabolic pathways has major functional consequences for tissues. In the low-oxygen conditions of the embryonic or damaged adult heart, the tissue relies on the glycolytic pathway to support organ function; whereas in the lungs of patients with ALI receiving ventilator treatment there is abundant oxygen, so upregulating the glycolytic pathway generates extra energy with which to protect lung tissue. In either case, HIF1α is involved in steering metabolic function in the appropriate direction. The findings of both papers suggest that enhancing glycolytic function could be protective in certain clinical scenarios—an idea that deserves attention and further testing.

**Eckle T, Brodsky K, Bonney M, Packard T, Han J, et al. (2013) HIF1A Reduces Acute Lung Injury by Optimizing Carbohydrate Metabolism in the Alveolar Epithelium. doi:10.1371/journal.pbio.1001665**

**Breckenridge RA, Piotrowska I, Ng K-E, Ragan TJ, West JA, et al. (2013) Hypoxic Regulation of** ***Hand1*** **Controls the Fetal-Neonatal Switch in Cardiac Metabolism. doi:10.1371/journal.pbio.1001666**

